# Women’s Attitudes Regarding Prenatal Testing for a Range of Congenital Disorders of Varying Severity

**DOI:** 10.3390/jcm3010144

**Published:** 2014-01-21

**Authors:** Mary E. Norton, Sanae Nakagawa, Miriam Kuppermann

**Affiliations:** 1 Department of Obstetrics, Gynecology and Reproductive Sciences, University of California, 350 Parnassus Ave, Suite 810, San Francisco, CA 94143-0705, USA; E-Mails: kuppermannm@obgyn.ucsf.edu (S.N.); nakagawas@obgyn.ucsf.edu (M.K.); 2 Kaiser Permanente, San Francisco, CA 94143, USA

**Keywords:** prenatal genetic screening, fragile X syndrome, spinal muscular atrophy, abortion, prenatal diagnosis

## Abstract

Little is known about women’s comparative attitudes towards prenatal testing for different categories of genetic disorders. We interviewed women who delivered healthy infants within the past year and assessed attitudes towards prenatal screening and diagnostic testing, as well as pregnancy termination, for Down syndrome (DS), fragile X (FraX), cystic fibrosis (CF), spinal muscular atrophy (SMA), phenylketonuria (PKU) and congenital heart defects (CHD). Ninety-five women aged 21 to 48 years participated, of whom 60% were Caucasian, 23% Asian, 10% Latina and 7% African American; 82% were college graduates. Ninety-five to ninety-eight percent indicated that they would have screening for each condition, and the majority would have amniocentesis (64% for PKU to 72% for SMA). Inclinations regarding pregnancy termination varied by condition: Whereas only 10% reported they would probably or definitely terminate a pregnancy for CHD, 41% indicated they would do so for DS and 62% for SMA. Most women in this cohort reported that they would undergo screening for all six conditions presented, the majority without the intent to terminate an affected pregnancy. These women were least inclined to terminate treatable disorders (PKU, CHD) *versus* those associated with intellectual disability (DS, FraX) and were most likely to terminate for SMA, typically lethal in childhood.

## 1. Introduction

With recent advances in molecular genetics, carrier screening and prenatal diagnosis is now available for a broad array of disorders. Screening is currently recommended in pregnancy for a number of genetic (single gene or Mendelian) disorders, chromosomal abnormalities and structural birth defects in the fetus [1,2,3,4]. It is generally agreed that the following criteria should be met for screening programs to be effective: (1) A disorder of sufficient severity to warrant screening; (2) A high frequency of carriers in the screened population; (3) The availability of an inexpensive and dependable test with low false-negative and false-positive results; (4) Access to genetic counseling for couples identified as carriers; (5) The availability of prenatal diagnosis; and (6) The acceptance and voluntary participation by the population targeted for screening [5].

In practice, disease incidence and severity and the availability of an effective screening test will often culminate in an expert opinion that population screening for a given disorder should be recommended, with little data collected regarding the attitudes of the target population. Despite increased recognition of the importance of involving patients in medical decisions [6,7,8], consideration of patient attitudes and preferences is not currently employed to determine prenatal genetic screening policies, an area that is particularly value-laden and where such considerations are arguably the most important. Another assumption underlying much of the discussion surrounding prenatal genetic testing is that screening tests should focus on disorders for which women would elect to terminate their pregnancy or which are severe enough that termination of pregnancy is considered a reasonable option by the medical profession. Whether the target population shares this assumption is unknown. While previous studies have retrospectively evaluated differences in the rate of termination of pregnancy for disorders of increased severity or those that include intellectual disability [9,10,11], few to no data exist on how women compare or value testing for different categories of conditions and whether they associate a desire for testing with an inclination toward termination for a given condition.

The objective of this project was to better understand women’s attitudes regarding prenatal genetic testing for a broad range of congenital disorders, including those that are potentially treatable, those that include substantial intellectual disability as a primary feature and those that are primarily medical disorders with a shortened life expectancy. In particular, the study sought to determine what women who had recently given birth would be inclined to do in their next pregnancy if they were offered the option of screening, diagnostic testing and termination of pregnancy for affected fetuses with a broad array of congenital disorders with varying characteristics.

## 2. Methods

Between September 2010 and May 2012, we interviewed Kaiser Permanente Northern California (KPNC) members aged 18–48 years old who had given birth within the past year. Kaiser Permanente Northern California (KPNC) is a large, integrated healthcare organization that provides care for more than 3.2 million residents, including approximately 35,000 pregnant women, per year. Except for the lowest and highest income earners, the KPNC membership is representative of the total population in the region. To recruit participants, letters were sent to all women who had given birth to healthy infants and who had had a postpartum visit at Kaiser San Francisco within the past 12 months. Although decision making regarding testing typically takes place during pregnancy, we recruited postpartum women due to concern about the potential for raising pregnant women’s anxieties about birth defects that might affect their infants, including those for which testing is not yet available or is not routinely offered.

Standard prenatal counseling at Kaiser involves the offer of screening for single gene disorders as recommended by the American College of Obstetricians and Gynecologists (ACOG), and a discussion and the offer of screening and diagnostic tests for aneuploidy, which are offered regardless of maternal age. Thus, all participants would have been offered screening for neural tube defects, Down syndrome and cystic fibrosis. Screening for fragile X (FraX) and spinal muscular atrophy (SMA) is not routinely offered, nor is specific screening for congenital heart defects (CHD), other than with nuchal translucency and routine second trimester anatomy ultrasound.

Our goal was to determine patient attitudes toward testing and termination for six congenital disorders of varying severity. The study included “prototype” conditions that reflected the types of disabilities for which testing is currently available or may become available in the future. The six disorders that were included (Down syndrome (DS), phenylketonuria (PKU), congenital heart disease (CHD), spinal muscular atrophy (SMA), fragile X syndrome (FraX) and cystic fibrosis (CF)) are common and were selected as they represent different characteristics with regards to severity, the presence of cognitive disability, the presence of physical abnormalities, the availability of treatment and life expectancy (Table 1).

Trained research assistants interviewed each participant individually. After signing informed consent, each participant completed a self- or interviewer-administered demographic/attitudinal survey instrument, which included questions regarding whether and how well they knew an individual with any of the six disorders under investigation. The conditions were then presented to the participant in random order, using a computerized tool. For each disorder, the name of the condition was provided along with a description of its characteristics (its effects on appearance, cognition and mental health, behavior, physical and medical health and the life expectancy), the prevalence of the condition and whether it was associated with maternal age and race/ethnicity.

Following the description of each disorder, the participants were asked whether they thought they would elect to have a blood (screening) test or an amniocentesis (diagnostic test) to detect the disorder and whether they would opt to terminate an affected pregnancy. Response options included “definitely” and “probably” would have, “uncertain” and “probably” and “definitely” would not have screening, diagnostic testing or a termination. For simplicity, participants were not asked to consider decisions regarding amniocentesis based on characteristics of screening tests, but rather to consider whether the disorder was severe enough to warrant diagnostic testing.

To assess general prenatal testing and abortion attitudes, participants were presented with a series of statements for which they were asked to indicate their agreement using a 5-point Likert scale ranging from “strongly disagree” to “strongly agree”. The “value of testing information” scale consisted of a single item: “Having prenatal testing is a way to make me less anxious during my pregnancy”. We assessed abortion attitude by asking about agreement with the statement: “Abortion should be generally available to any woman who wants one”.

We conducted descriptive analyses to profile the sample, including the examination of means and proportions, measures of variability and confidence intervals around these estimates. Approval was obtained from the Kaiser Permanente Institutional Review Board (IRB) for the study.

**Table 1:** Disorders and key characteristics presented to participants.

Disorder	Main features	Treatment *
Down syndrome	ID, typical facial features, medical issues; life expectancy, 60 s	As needed; none for ID
Fragile X	ID, facial features, behavioral/autistic issues; normal life expectancy	None
PKU	Metabolic disorder, ID without rx, good outcome with treatment, rx somewhat time consuming/intense; normal life expectancy	Dietary
CHD	Outcomes vary, usually no other abnormalities; somewhat shortened to normal life expectancy	Surgical
SMA	Severe hypotonia, respiratory failure; death by age 2	None
Cystic fibrosis	pulmonary/pancreatic dysfunction, significant medical problems; life expectancy, late 30 s	Medical

* Refers to medical treatment, as opposed to educational interventions; ID = intellectual disability; PKU = phenylketonuria; CHD = congenital heart defect; SMA = spinal muscular atrophy; rx = treatment.

## 3. Results

We recruited and interviewed 95 women who had delivered healthy infants within the previous year. Participants ranged from 21 to 48 years of age and were racially and ethnically diverse; 60% were Caucasian, 23% were Asian, 10% were Latina and 7% were African American. From a socio-economic perspective, they were less diverse, with most having college degrees, an income >$100,000/year and being employed and married or living with a significant other. For 39%, the recent pregnancy was their only prior pregnancy (Table 2). In all, 27 (28%) reported having had one or more abortions.

In terms of their familiarity with the genetic conditions of interest, more than half reported knowing someone with Down syndrome and 12% indicated that they had a relative with the condition. Nearly half (45%) reported knowing someone with a congenital heart defect, 13% knew someone with cystic fibrosis and 27% reported knowing someone with another congenital or genetic disorder. These ranged from minor conditions, such as color blindness, to a range of genetic disorders, such as hemophilia and sickle cell disease. Relatively few subjects knew or were related to anyone with SMA, PKU or fragile X syndrome (Table 3).

**Table 2:** Sociodemographic characteristics of the population.

Characteristic	*N* (%)
**Maternal age**	
<35 years	49 (51.6)
≥35 years	46 (48.4)
**Race/Ethnicity**	
Caucasian	57 (60.0)
Asian	22 (23.2)
Latina	9 (9.5)
African-American	7 (7.4)
**Gravidity**	
1	37 (38.9)
2	31 (32.6)
>2	27 (28.5)
**Living children**	
1	59 (62.1)
>1	36 (37.9)
**Relationship status**	
Married	85 (89.5)
Significant other	5 (5.3)
Single	5 (5.3)
**Education**	
High school	2 (2.1)
Some college	15 (15.8)
College degree	29 (30.5)
Postgraduate	49 (51.6)
Employed	73 (76.8)
**Yearly income**	
<$25,000	7 (7.4)
$25–50,000	9 (9.6)
$50–100,000	23 (24.5)
$100–150,000	32 (34.0)
>$150,000	23 (24.5)

**Table 3:** The number of subjects who are related to or know someone with congenital disorders.

Disorder	Relative	Know someone	Know moderately/very well
	*N* (%)	*N* (%)	*N* (%)
Down syndrome	11 (12)	53 (56)	29 (31)
Cystic fibrosis	0	12 (13)	7 (7)
Fragile X	0	4 (4)	3 (3)
SMA	1 (1)	7 (7)	5 (5)
PKU	1 (1)	4 (4)	2 (2)
CHD	9 (9)	43 (45)	30 (32)
Other *	11 (12)	26 (27)	24 (25)

SMA = spinal muscular atrophy; PKU = phenylketonuria; CHD = congenital heart defect; * A broad range of other inherited or congenital disorders, such as hemophilia, color blindness and spina bifida.

With regard to personal experience with testing, 98% reported having had testing for Down syndrome, 37% had had testing for cystic fibrosis, 21% reported testing for Tay-Sachs disease and 16% had had an Ashkenazi Jewish panel. Fewer than 10% had had testing for fragile X syndrome or spinal muscular atrophy.

In exploring attitudes toward prenatal testing and pregnancy termination, 81% of the participants reported somewhat or strongly agreeing with the statement, “Having prenatal testing is a way to make me less anxious during my pregnancy”. In addition, 85% somewhat or strongly agreed with the statement, “Abortion should be generally available to any woman who wants one”.

**Figure 1:**
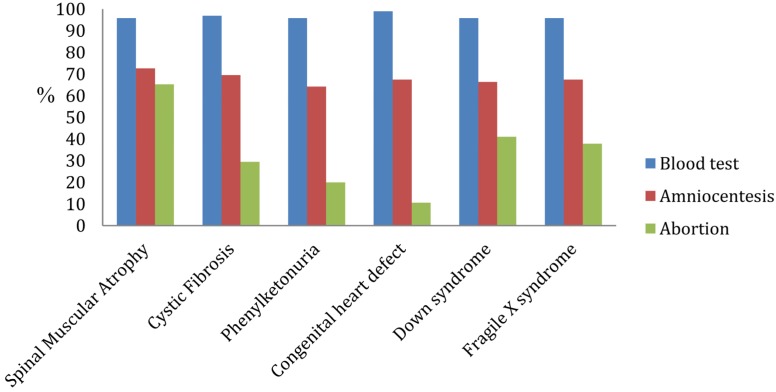
Proportion of patients indicating they would definitely or probably undergoing screening (blood test), diagnostic testing (amniocentesis) or pregnancy termination for each condition.

Most participants indicated that they would opt to have a screening test for each of these conditions (95%–98%, depending on the specific test), and the majority also indicated that they would have amniocentesis (ranging from 64% for PKU to 73% for SMA). Inclinations regarding pregnancy termination varied much more substantially by condition: While only 11% of the participants indicated that they would choose to terminate a pregnancy for a congenital heart defect, 41% would be inclined to do so for Down syndrome and 65% for SMA (Figure 1). While the number of subjects that were moderately or very familiar with these conditions was relatively small, knowing someone with CF and SMA was associated with an increased likelihood of having had testing for the disorder (*p* = 0.002 for CF and *p* = 0.01 for SMA). This was despite the fact that these were unrelated acquaintances, rather than related individuals, which would indicate an increased risk that our subject would be a carrier of the condition. The association of familiarity with an affected individual and testing for the condition was not found with the other disorders.

## 4. Discussion

While over 95% of women in this cohort indicated that they would choose to undergo screening for all of the conditions presented to them, the majority would do so without intent to terminate an affected fetus. Women were less inclined to terminate treatable disorders, such as PKU and congenital heart defects, as compared to those associated with intellectual disability (Down syndrome and fragile X syndrome). Patients were most inclined to terminate for SMA, which is typically lethal in childhood. Only for SMA would more than half of women terminate their pregnancy; for all of the other disorders presented, even those who would choose diagnostic testing did not feel they were likely to terminate based on the results. This represents a substantially different view of the purpose of prenatal genetic testing than what is typically discussed by the medical community; both advocates and critics of prenatal genetic testing commonly describe the goal as identifying affected fetuses with an intent to abort those that are abnormal. In this study, most patients clearly did not view the purpose of such testing as identifying those that should be terminated.

Few previous studies have investigated the attitudes of patients regarding screening or testing for different categories of disorders, although some studies have reported results similar to this one with regard to differences in the uptake of screening, diagnostic testing and pregnancy termination. A study in the UK focusing on 109 relatives of individuals affected with CF found that while most (75%) would undergo screening for this disorder, fewer than half would have a pregnancy termination [12]. Another study of the parents of children with CF found that while most would have a prenatal diagnosis, the decision to have an abortion was far more difficult [13]. In a study conducted to gauge consumers’ opinions towards genetic testing for diseases and enhancements, patients were asked to indicate traits and conditions for which they would choose reproductive genetic testing. Most, but not all, respondents were pregnant women intending to have prenatal diagnostic procedures. The majority indicated that they would elect to have prenatal genetic testing for mental retardation (75%), deafness (54%), blindness (56%), heart disease (52%) and cancer (51%); 49.3% would choose testing for a condition that resulted in death by five years of age. Those findings were similar to the results in this report, although these investigators did not distinguish between screening and diagnostic testing and did not assess whether the desire for reproductive testing was associated with the intent to terminate an affected pregnancy [14].

It appears that some disparity exists between prospective parents and their care providers. During pregnancy, prospective parents seek reassurance that their fetus is developing normally. Providers, on the other hand, frequently see the purpose of prenatal genetic testing as identifying abnormalities for which the patient would choose to terminate a pregnancy. In a review of reproductive genetic counseling, Pergament *et al.* noted that “Decisions made after genetic counseling have limited parameters: parents must consider diagnostic testing after positive screening for a genetic disorder and then either continue the pregnancy or electively terminate, if the pregnancy is affected” [15]. Many providers, as part of the conversation about the choice to undergo testing, recommend against it for disorders if the patient indicates that she is unlikely to elect an abortion if the specific abnormality is identified.

In a systematic review of reproductive genetic testing, Green *et al.* note that “Clearer consensus is required about the knowledge that is necessary and sufficient for women to have when they are making decisions about prenatal and newborn genetic testing [16]. Put more bluntly, what is it that people do need to know and whose business is it to decide that? Professionals have been preoccupied with conveying certain kinds of information (e.g., procedural matters, risk estimates) but have virtually ignored others (e.g., what it might be like to bring up a child with the condition in question), and an approach based on parents’ needs rather than staff needs is long overdue” [16]. This study is a step toward collecting data to improve and inform such an updated approach.

The study is not without limitations. The sample size was relatively small, and the participants were all recruited from one Kaiser site in Northern California. While the cohort was racially and ethnically diverse, for the most part, they were highly educated. The intent originally was to use generic descriptions of the conditions to avoid prior perceptions of the significance of these disorders. However, in focus groups conducted prior to the study, it was evident that many women quickly recognized the various disorders, so ultimately, the conditions were labeled; the participants may therefore place value on each condition that has been colored by prior experience, associations or preconceptions. In addition, the study was based on hypothetical situations, and decisions regarding pregnancy termination might be quite different when women are presented with an actual diagnosis of an affected pregnancy compared to the hypothetical situation posed in the study.

## 5. Conclusions

While the field of genetics, and prenatal genetics in particular, has evolved at a remarkable rate, the approach to the assessment of how to best implement new tests and technologies has lagged. The field has continued with a paradigm of offering individual tests for single conditions, with the assumption that the primary outcome to consider is pregnancy termination. It appears from this study that the primary consideration of this cohort of patients is quite different and that there is value to information about fetal disorders that extends beyond a decision regarding pregnancy continuation or termination.
